# Nitrogen Addition and Warming Independently Influence the Belowground Micro-Food Web in a Temperate Steppe

**DOI:** 10.1371/journal.pone.0060441

**Published:** 2013-03-27

**Authors:** Qi Li, Huahua Bai, Wenju Liang, Jianyang Xia, Shiqiang Wan, Wim H. van der Putten

**Affiliations:** 1 State Key Laboratory of Forest and Soil Ecology, Institute of Applied Ecology, Chinese Academy of Sciences, Shenyang, China; 2 State Key Laboratory of Vegetation and Environmental Change, Institute of Botany, Chinese Academy of Sciences, Beijing, China; 3 Netherlands Institute of Ecology, Terrestrial Ecology Department, Wageningen, The Netherlands; 4 State Key Laboratory of Cotton Biology, Henan Key Laboratory of Stress Plant Biology, School of Life Sciences, Henan University, Henan, China; 5 Laboratory of Nematology, Wageningen University and Research Centre, Wageningen, The Netherlands; UC Irvine, United States of America

## Abstract

Climate warming and atmospheric nitrogen (N) deposition are known to influence ecosystem structure and functioning. However, our understanding of the interactive effect of these global changes on ecosystem functioning is relatively limited, especially when it concerns the responses of soils and soil organisms. We conducted a field experiment to study the interactive effects of warming and N addition on soil food web. The experiment was established in 2006 in a temperate steppe in northern China. After three to four years (2009–2010), we found that N addition positively affected microbial biomass and negatively influenced trophic group and ecological indices of soil nematodes. However, the warming effects were less obvious, only fungal PLFA showed a decreasing trend under warming. Interestingly, the influence of N addition did not depend on warming.

Structural equation modeling analysis suggested that the direct pathway between N addition and soil food web components were more important than the indirect connections through alterations in soil abiotic characters or plant growth. Nitrogen enrichment also affected the soil nematode community indirectly through changes in soil pH and PLFA. We conclude that experimental warming influenced soil food web components of the temperate steppe less than N addition, and there was little influence of warming on N addition effects under these experimental conditions.

## Introduction

Climate warming has been predicted to increase the global surface temperature by 1.8–4.0°C at the end of this century [1]. The rise in temperature could have profound effects on terrestrial ecosystems, such as changes in competition between species [2], altering plant productivity [3], [4], and in turn, influencing the supply of carbohydrates to belowground subsystems through root growth [5]. In addition to climate change drivers, terrestrial ecosystems are also affected by other global change phenomena, such as nitrogen deposition. It is predicted that global deposition of reactive N to the environment will increase from 100 Tg N yr^−1^ (in 1995) to 200 Tg N yr ^−1^ by 2050 [6]. The intensive alteration of global nitrogen (N) cycles due to anthropogenic activities could change plant species composition and community structure [7], [8], [9], with consequent impacts on the structure and functions of soil ecosystems. Although individual effect of warming and N enrichment on ecosystem functioning has received wide attention [10], [11], [12], their combined effects are still unknown [13], especially on the responses of belowground organisms [13], [14], [15], [16], [17].

The few observations on the interactive effects of warming and N addition are relatively inconsistent. For example, there were additive effects of N addition and winter warming on plant productivity and soil N availability in temperate old fields [18], [19], and N addition increased the temperature sensitivity of the slowly cycling soil C pool in tropical forest [20]. In the Harvard Forest Long Term Ecological Research Site (LTER), the N availability diminished the warming effect on soil respiration in autumn [21], [22]. In a subarctic heath ecosystem, warming was found to negate the N addition effect on plant and microbial biomass after fifteen years of climate change manipulations [23]. On the other hand, N deposition and climate warming influenced litter decomposition and the associated microbial communities independently in low-alpine heath [24]. Given the important roles of soil biota in terrestrial ecosystems [15], understanding the direction and magnitude of interactive effects of N enrichment and warming on soil food webs and their components is crucial for predicting the changes in ecosystem structure and functioning under global climate change.

N addition and warming can each alter the activity of microbial decomposers, influencing the quantity of C lost from soils via respiration, and the transport of C from the surface into soils as dissolved organic C [25], [26]. N deposition can directly change soil C-cycling rates by inhibiting the microbial production of ligninolytic enzymes and enhancing cellulolytic enzyme activity [27]. Moreover, N deposition can also influence soil microorganisms and decomposition processes indirectly through altering plant composition and productivity by alleviating nitrogen limitation of plant growth [28]. Altogether, warming and N deposition can alter the rates of heterotrophic microbial metabolism in soil, and consequently the flow of C and N through soil food webs. While both warming and N deposition can impact soil biogeochemical processes, most of the research to date only quantified these effects independently, and the majority of these studies have focused on aboveground subsystems [10], [29]. Until now, there is relatively little knowledge on how different global change drivers interactively influence soil food web composition and functioning [15], [17], [30]. Since soil biota may influence biogeochemical cycling and physical conditions in terrestrial ecosystems, their responses to global changes are important at the ecosystem scale [15], [31]. Recent reviews of global change effects on soil biota also suggested that there is an urgent need for long-term studies to investigate interactions between different agents acting in concert [32], [33].

As part of the Eurasian grassland biome, the temperate steppe in northern China has suffered from over-grazing since 1980s, resulting in severe land degradation and soil N deficiency [34]. In addition, temperature in these areas increased substantially during past half century [35], and warming could stimulate evaportanspiration and reduced soil water availability, consequently aggravating soil water deficits in the semiarid regions [36]. Thus, it is predicted that the grassland ecosystems in this region are sensitive to climate change [37], [38]. However, there has been little information on how belowground biota respond to climatic change in this region, particularly to the combined driving factors, i.e. climate warming and N deposition. Therefore, a field experiment has been set up to examine the effects of climate warming, atmospheric N deposition, and their interactions. Our objectives were to explore the respective and interactive effects of N addition and warming on the micro-food web in a temperate steppe of Northern China. We focused on microbial biomass and nematode trophic groups as important components of soil micro-food web, because the micro-food web governs nutrient cycling and mineralization processes, and strongly determines the responses of belowground subsystem to climate change [15]. Previous studies at the field site we conducted our research have demonstrated that warming negatively affected root production [5], soil respiration, and microbial biomass [39]. In addition, N addition was found to stimulate gross ecosystem productivity and ecosystem C exchange by increasing C assimilation in the temperate steppe [26]. Accordingly, where they occur separately, warming and N addition appear to have an opposite effect on soil C dynamics [21]. Based on these previous studies and other reports [20], [33], [40], we hypothesized that N enrichment will positively affect micro-food web components, while warming will negatively influence them. Further, we hypothesized that warming and N addition will be counteracting each other, so that their interaction effect will be neutral. Structural equation modeling (SEM) was used to test whether N addition or warming influenced the components of soil food web directly or indirectly through changes in soil abiotic characters and/or plant growth.

## Materials and Methods

### Study site

This study was conducted at a field site of the Duolun Restoration Ecology Experimentation and Demonstration station of the Institute of Botany, the Chinese Academy of Sciences. The station is located at a temperate steppe in Duolun County, Inner Mongolia (42°02′N, 116°17′E, 1324ma.s.l), China. The soil in the study site is Chestnut soil (Chinese classification), or Haplic Calcisols according to the FAO classification, with sand, silt, and clay being 62.7%, 20.3%, and 17.0%, respectively. Mean bulk density is 1.31 g·cm^−3^ and pH is 7.7. The local climate in our study area has increased 0.45 °C over the past half century (1953–2005) [35]. N deposition in 2005–2006 was estimated at about 20 kg ha^−1^ yr^−1^ in this region [34]. The dominant plant species are *Stipa krylovii* Roshev., *Artemisia frigida* Willd., *Potentilla acaulis* L., *Cleistogenes squarrosa* (Trin.) Keng., *Allium bidentatum* Fisch. ex Prokh., *and Agropyron cristatum* (L.) Gaertn. Traditional land uses in this region include livestock grazing and farming. From the late 1950s to the 1970s, the government put strict policies into effect to ban grazing. Since the economic reforms and open-door policy in 1978, lands were thrown open to private use. After 2000, new bans on grazing have been carried out by the local government, but cattle and sheep grazing are still conducted in some areas of this region.

### Experimental design

The experiment consisted of a complete random block design with six treatments, of which we included four treatments in the present study. There were six replicates of each treatment. Thirty-six plots of 3 m×4 m were arranged in a 6×6 matrix. The distance between two adjacent plots was 3 m. One of the six plots in each row was randomly assigned to one of the six treatments. In our study, we examined effects of control (CK), diurnal (24 h) warming (W), N addition (N), and diurnal warming plus N addition (WN) treatments. Effects of day and night warming were also included in the experimental design, but these treatments were not evaluated in the present study.

All the warmed plots were heated continuously by MSR-2420 infrared radiators (Kalglo Electronics Inc., Bethlehem, PA, USA) suspended 2.25 m above the soil surface. Across the four growing seasons from 2006 to 2009, warming treatment significantly increased the soil temperature at the depth of 10 cm by 1.79 °C. More detailed information of warming treatments on soil microclimates can be found in [41]. In each control or N addition plot, one ‘dummy’ heater with the same shape and size as the infrared heater was installed as in the heated plots to simulate the shading effects of the infrared radiator. All the heaters of the warming treatments were set at a radiation output of approximately 1600 W. The warming treatment started on 23 April 2006. N additions were spread by hand before the first rain event in the rainy season, and were applied once a year with NH_4_NO_3_ in the form of pellets. Given that N effects on species composition and ecosystem production saturate at N addition rates of about 10.5 g N m^−2^ yr^−1^ in this region [37], the level of N addition in this study was 10 g N m^−2^ yr^−1^. The experiment was started in 2006 and soil samples were collected in September 2009 and 2010. From each plot, five soil cores of 2.5 cm diameter were collected from a depth of 0–15 cm below soil surface and stored at 4°C until further analysis. In late August of each year, we clipped a 1×1 m^2^ quadrat in each subplot. Aboveground and belowground biomasses were measured. Root biomass at 0–15 cm depth was measured by soil auger (8 cm in diameter). The soil was carefully removed from the root system and the roots were thoroughly rinsed. The dry masses of aboveground and belowground biomass were determined by over-drying at 70 °C to constant weight.

### Soil analyses

**Figure 1:**
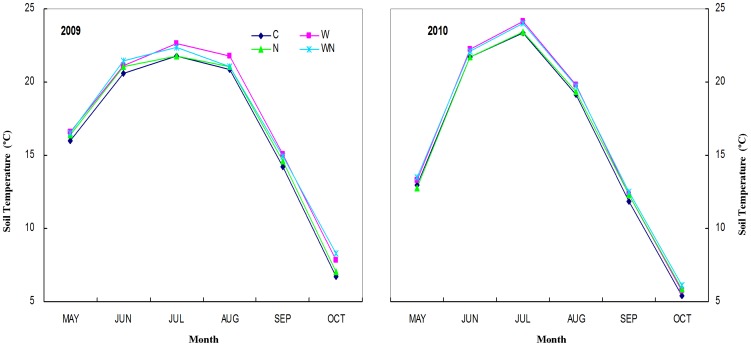
Monthly mean soil temperature in 2009 and 2010 as affected by nitrogen addition and warming in a temperate steppe.

Soil temperature at the depth of 10 cm was recorded automatically with a Datalogger (STM-01 Soil Temperature Measurement System; Henan Electronic Institute, Zhengzhou, China). Six soil temperature measurements in each plot were collected with 10-min intervals and averages of the six measurements were stored as the hourly averages. The average soil temperature of each month is presented in (Fig. 1). The total soil carbon (SOC) and nitrogen (TN) were determined by a TruSpec CN Elemental Analyzer (Leco Corporation, USA). Soil pH was determined with a glass electrode in 1∶2.5 soil:water solution (w/v). Soil moisture (SM) was measured by weight loss after drying at 105°C for 48 h.

### PLFA analysis

The soil microbial community was characterized using phospholipid fatty acid (PLFA) analysis as described by Bossio & Scow (1998) with slight modifications [42]. PLFA was extracted from 8 g freeze-dried soil, and the extracted fatty acid methyl esters were identified with a standard qualitative mix ranging from C9 to C30 and a MIDI peak identification system (Microbial ID. Inc., Newark, DE).

The sum of the following PLFAs was used as the measure of bacterial biomass: i15∶0, a15∶0, 15∶0, i16∶0, 16∶1ω7c, i17∶0, a17∶0, 17∶0, cy17∶0, and cy19∶0. The fatty acid 18∶2ω6 and 18∶1ω9c were used as indicator of fungal biomass [43] and 16∶1ω5c for AM fungi (AMF) [44], [45]. Taken together, all of the PLFAs indicated above were considered to be representative of the total PLFAs of soil microbial community.

### Nematode community analysis

Nematodes were extracted from 100 g of soil (fresh weight) by a modified cotton-wool filter method [46], [47]. Nematode populations were expressed as number of nematodes per 100 g dry soil, and at least 150 nematodes from each sample were identified to genus level using an inverted compound microscope. The nematodes were assigned to the following trophic groups characterized by feeding habits: (1) bacterivores (Ba); (2) fungivores (Fu); (3) omnivore-predators (Om-Ca) and (4) plant parasites (PP) following Yeates et al. (1993) [48].

The following nematode community indices were calculated:

1. Trophic diversity TD = 1/Σp_i_^2^; where p_i_ is the proportion of trophic group i;
2. Generic richness SR = (S−1)/ln(N), where S is the number of taxa and N is the number of total nematodes [49];
3. Maturity index MI = ∑v(i)×f(i), where v(i) is the c-p value of taxon i according to their *r* and *K* characteristics following Bongers (1990) [44], f(i) is the frequency of taxon i in a sample;
4. Plant parasite index PPI = ∑v(i)×f(i), which was determined in a similar manner with maturity index for plant parasitic genera [50];
5. Structure index SI = 100×(∑k_s_n_s_/(∑k_s_n_s_+∑k_b_n_b_));
6. Enrichment index EI = 100×(∑k_e_n_e_/(∑k_e_n_e_+∑k_b_n_b_)),

where k_b_ is the weight assigned to guilds Ba2 and Fu2 and n_b_ is the abundance of nematodes in guilds Ba2 and Fu2, which indicate basal characteristics of the food web; k_s_ the weight assigned to guilds Ba3–Ba5, Fu3–Fu5, Om4–Om5 and Ca2–Ca5, n_s_ is the abundance of nematodes in these guilds, which represent the structure condition of the food web; k_e_ the weight assigned to guilds Ba1 and Fu2, and n_e_ is the abundance of nematodes in these guilds, which represent an enriched condition of the food web [51]. Bax, Fux, Cax, Omx, (where x = 1–5) represent the functional guilds of nematodes that are bacterivores, fungivores, predators and omnivores where the guilds have the characters indicated by x on the colonizer-persister (cp) scale (1–5) following Bongers & Bongers (1998) [52].

### Statistical analysis

Nematode abundances and the PLFA-derived biomass were ln(x+1) transformed prior to statistical analysis. General linear model analysis of variance was used to test the main effects and interactions of date, N addition and warming on soil properties and biota. The least significant difference (LSD) test was used when the treatment effects were significant. All statistical analyses were performed by SPSS statistical software (SPSS Inc., Chicago, IL). Difference at *P*<0.05 level was considered to be statistically significant. For the PLFA and nematode data, no interactive effects with sampling dates were observed in the ANOVA analysis, so the data were combined and presented for the average of two years.

Structural equation modeling (SEM) was used to investigate how N addition and warming affected soil microbe and nematode trophic groups in each year. We started the SEM procedure with an initial SEM model, based on our predictions [53]. In this model we hypothesized that N addition and climate warming may alter soil abiotic properties and plant biomass, which in turn may affect microbial community and soil nematode trophic groups. Soil abiotic characteristics, microbial community and nematode community were treated as latent variables [54]. We used soil moisture,soil organic C, total nitrogen and soil pH as the indicators of soil abiotic characteristics; bacterial, fungal and AMF PLFA as the indicators of microbial community; and the abundance of nematode trophic groups as the indicators of nematode community. In the initial model, the relationships between plant biomass and micro-food web were not significant and contributed little for the fit of the model, so plant biomass was removed from the final model for simplification. In the SEM analysis, we compared the model-implied variance-covariance matrix against the observed variance-covariance matrix with maximum likelihood estimation. We used the χ^2^ and its associated *P*-value to judge the model fit to the data. In the first phase, we constructed models containing all soil abiotic variables proposed to have influences on soil micro-food web. Based on the results of goodness-of-fit tests, we excluded less predictive measures (soil moisture, soil organic carbon and total nitrogen) and retained soil pH as the most informative abiotic variables in the final model. By stepwise removal of non-significant paths from the model we selected the model that fit our data best. SEM analyses were performed using AMOS 5.0 (Amos Development, Spring House, Pennsylvania, USA).

## Results

### Soil charateristics and plant biomass

Although soil characteristics (SM and pH) and plant biomass varied between 2009 and 2010 (Table 1), no warming effects were observed on the tested parameters. Soil pH was decreased by 8% and 1% in 2009 and 2010, respectively, following N addition (*P*<0.01).

**Table 1:** Soil characteristics and above and belowground plant biomass as affected by nitrogen addition (N) and warming (W) in a temperate steppe (Means ± SD).

Date		SM (%)	pH	TN (g kg^−1^)	SOC (g kg^−1^)	A-Biomass (g m^−2^)	B-Biomass (g m^−2^)
2009	CK	4.58±0.87	7.20±0.09A	1.94±0.13	23.81±1.75	58.44±6.38	120.65±55.31
	W	4.05±0.34	7.29±0.10A	1.87±0.27	23.80±4.07	55.50±10.19	101.90±36.70
	N	4.31±0.61	6.68±0.14B	1.81±0.19	22.55±2.52	54.22±12.97	113.89±47.03
	WN	4.19±0.37	6.62±0.17B	1.79±0.17	22.94±1.63	50.27±10.89	184.89±100.20
2010	CK	3.13±0.60	7.42±0.17A	1.78±0.42	22.30±3.31	120.75±11.65	971.88±422.08
	W	3.13±0.74	7.29±0.18A	1.76±0.19	20.40±2.66	116.54±13.86	875.25±303.15
	N	3.47±0.67	7.31±0.08A	2.01±0.56	26.14±7.34	126.74±5.97	1199.58±269.78
	WN	3.02±0.42	7.25±0.08A	1.99±0.24	27.69±5.51	142.85±11.29	977.95±295.41
ANOVA (*P* values)							
Date		**	**	ns	ns	**	**
W		ns	ns	ns	ns	ns	ns
N		ns	**	ns	ns	ns	ns
W×N		ns	ns	ns	ns	ns	ns

Notes: ** indicates significant difference at *P*<0.01; ns indicates no significant difference. Capital letters indicate significant difference among different treatments. A-Biomass, aboveground plant biomass; B-Biomass, belowground plant biomass.

### Soil microbial community

**Figure 2:**
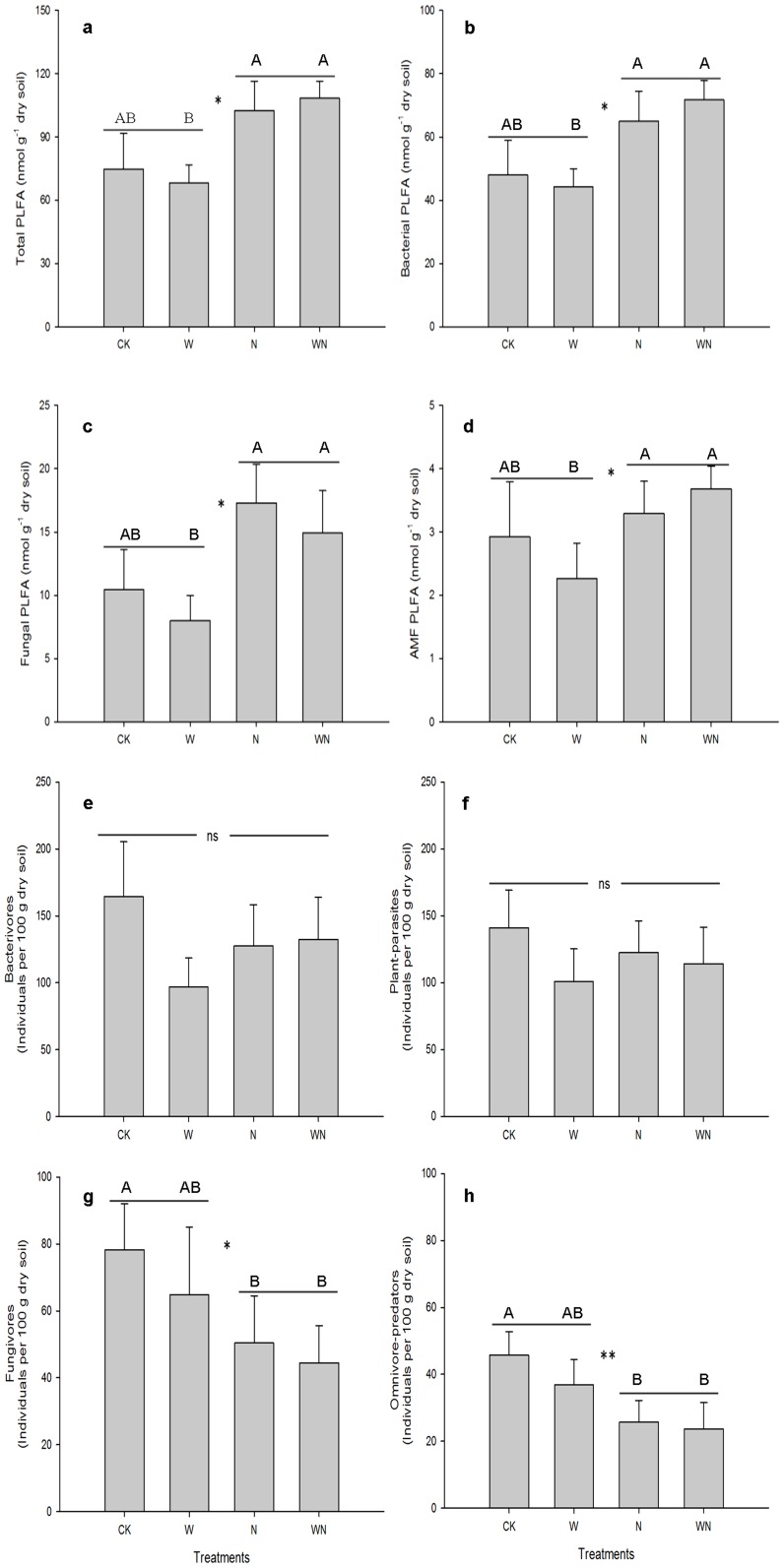
The PLFA biomasses for total (a) and indicator subgroups (b, c, d) and the abundance of nematode trophic groups (e–h) as affected by nitrogen addition and warming in a temperate steppe soil (Means ± **SE).** Bars indicate standard errors. Horizontal lines indicate the N treatment effects derived from General linear model analysis of variance. * and ** indicate N treatment effects significant at *P*<0.05 and *P*<0.01, respectively. Capital letters indicate significant differences among different treatments derived from LSD.

No sampling date effect was observed in the total and subgroup of PLFA biomass (Table 2). N addition effects on the PLFA biomass were more obvious than the warming effects. The average biomasses of total PLFA and the bacterial, fungal and AMF PLFA were enhanced 47.4%, 48.1%, 74.5% and 34.3%, respectively, by N addition (Fig. 2 a, b, c, d; *P*<0.05). Only fungal PLFA responded to warming, which resulted in lower fungal biomass under warming (Fig. 2c; *P*<0.05).

**Table 2:** Results (*P*-values) of ANOVA on the effects of Date, warming (W), nitrogen addition (N) and their interactions on the PLFA biomass, nematode trophic groups and ecological indices.

	PLFA				Trophic groups				Ecological indices					
	Total	Bacterial	Fungal	AMF	BF	FF	PP	OP	TD	SR	MI	PPI	EI	SI
Date	ns	ns	ns	ns	*	ns	ns	ns	*	ns	ns	ns	**	ns
W	ns	ns	*	ns	ns	ns	ns	ns	ns	ns	ns	ns	ns	ns
N	*	*	*	*	ns	*	ns	**	*	**	**	ns	ns	**
W×N	ns	ns	ns	ns	ns	ns	ns	ns	ns	ns	ns	ns	ns	ns

Notes: * and ** indicate significant differences at *P*<0.05 and *P*<0.01, respectively; ns indicates no significant difference. Total, total PLFA biomass; Bacterial, Bacterial PLFA; Fungal, Fungal PLFA; AMF, AMF PLFA; BF, bacterivores; FF, fungivores; PP, plant-parasites; OP, omnivore-predators; TD, trophic diversity; SR, generic richness; MI, maturity index; PPI, plant-parasite index; EI, enrichment index; SI, structural index.

### Soil nematode community

**Figure 3:**
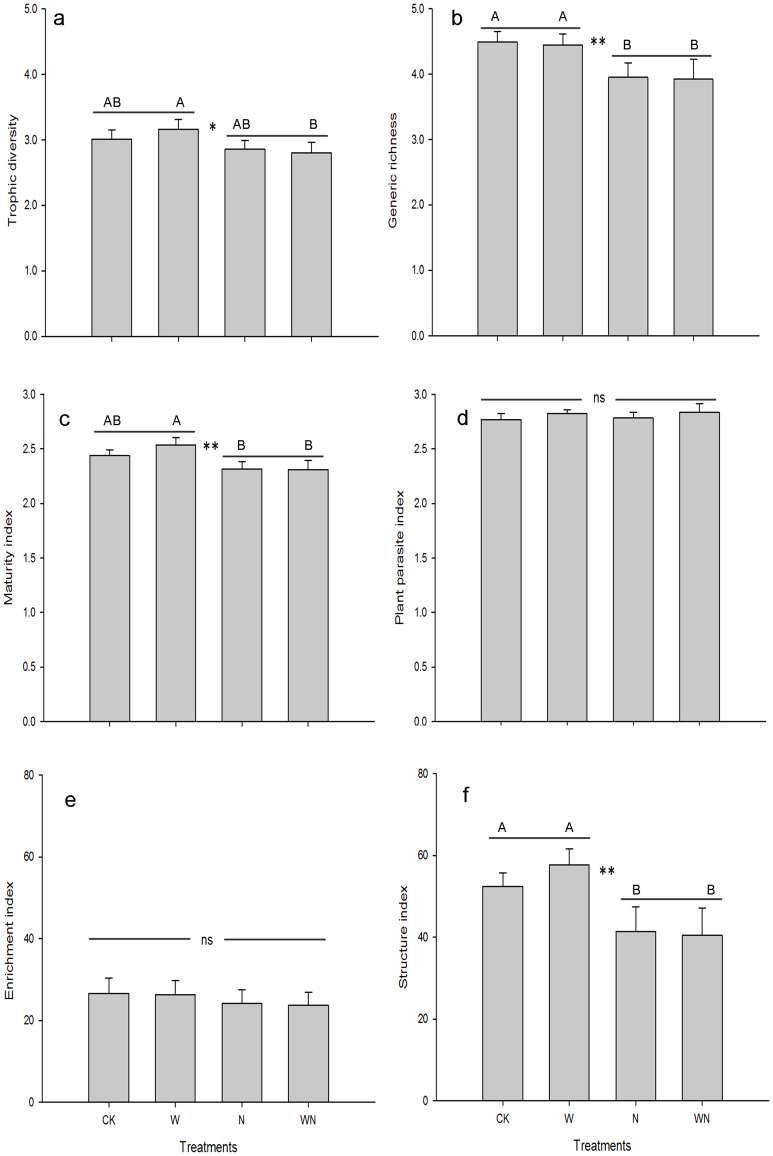
Nematode ecological indices as affected by nitrogen addition and warming in a temperate steppe soil (Means ± **SE).** Bars indicate standard errors. * and ** indicate N treatment effects significant at *P*<0.05 and *P*<0.01, respectively. Capital letters indicate significant differences among different treatments derived from LSD.

Warming and N addition didn't influence the total nematode abundance (data not shown). No warming effects were observed on nematode trophic groups and ecological indices. Among nematode trophic groups, only bacterivores varied between different sampling years (*P*<0.05), but no treatment effects were observed on the abundance of bacterivores and plant parasites (Fig. 2 e, f). Surprisingly, although the fungal PLFA was increased by N addition, the abundances of fungivores and omnivore-predators showed opposite patterns, as N addition reduced their abundances with 33.7% and 40.3%, respectively (Fig. 2 g, h; *P*<0.05). Among nematode ecological indices, trophic diversity, generic richness, maturity and structural indices were reduced by N addition (*P*<0.05), whereas plant-parasite index and enrichment index were not affected by the experimental treatments (Fig. 3).

### SEM results

**Figure 4:**
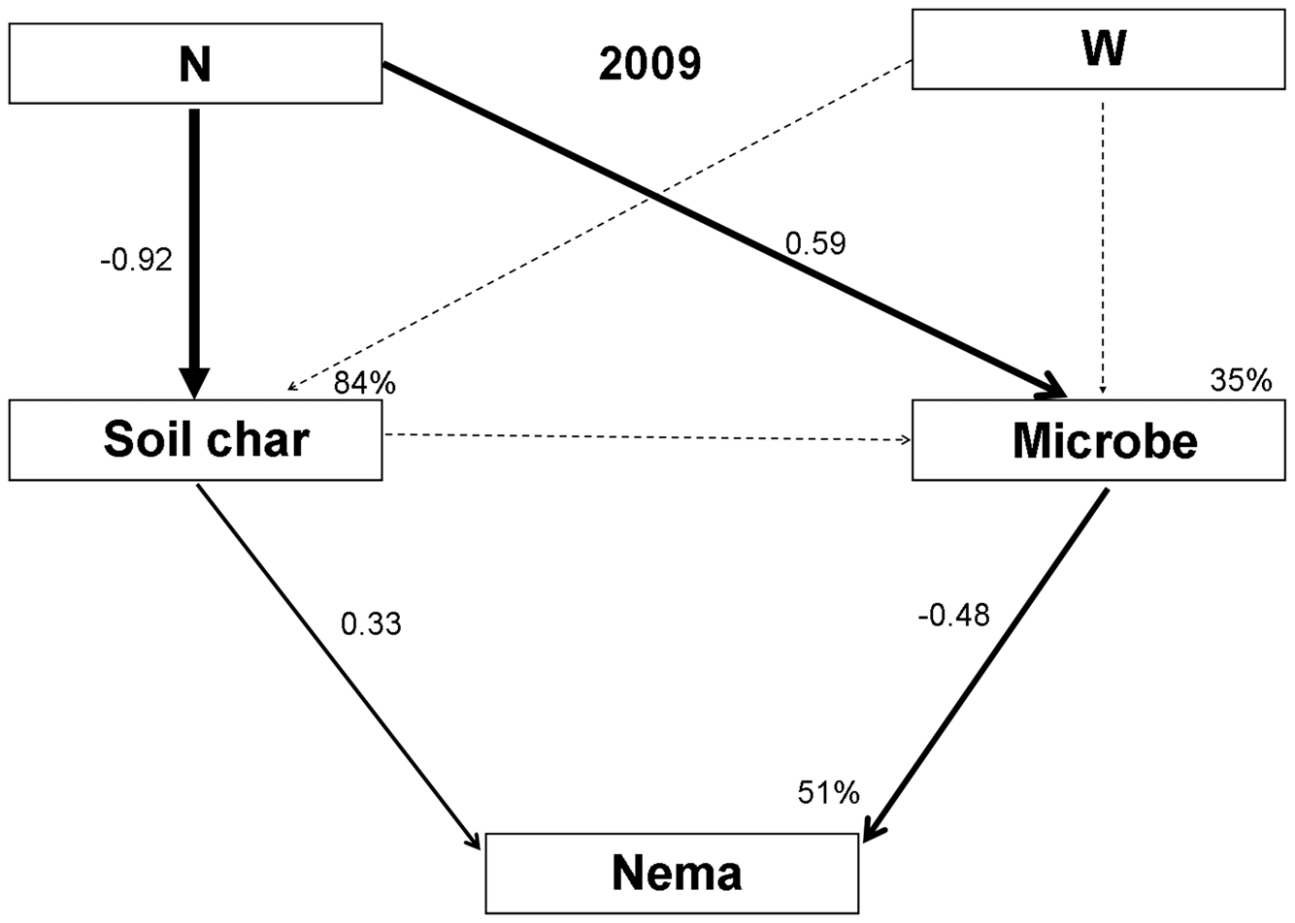
Structural equation models of N addition and warming effects on soil micro-food web components in a temperate steppe in 2009 (*χ^2^* = 27.511; df = 24, *P* = 0.281; CFI = 0.975; RMSEA = 0.080). Numbers on arrows are standardized path coefficients. Width of the arrows indicates the strength of the causal influence (non-significant pathways are dashed). N represents N addition effects; W, warming effects; Soil Char, soil characteristics; Microbe, soil microbial PLFA biomass; and Nema, soil nematode trophic groups.

Since no significant path way was observed in the final model of 2010, we only presented the SEM results of 2009. SEM analysis of 2009 data suggested that N addition altered soil nematode community composition indirectly through changes in soil microbial biomass (PLFA) and soil pH. However, warming effects were less obvious than those of N addition. The final models adequately fit the data on soil food web components in our study (standardized path coefficients are given in Fig. 4). The model of 2009 explained 35% of the variance in soil microbial biomass and 51% of variance in nematode abundance. The final SEM models indicated that soil food web components were stronger influenced by N addition directly rather than indirectly through changes in soil abiotic parameters (soil pH).

## Discussion

### N addition effects on soil micro-food web

In the present study, N addition enhanced microbial biomass, but negatively affected soil nematode trophic groups and community composition. The latter effect did not support our hypothesis. Low-levels of N addition have been reported to stimulate microbial growth by ameliorating N and C limitation through improving soil N availability and stimulating plant growth and litter decomposition [55]. However, the increase in microbial biomass did not lead to an increase in the abundance of nematode trophic groups. The differential responses of microbial biomass and nematode community composition might be driven by the complex interactions between bottom-up (resource quantity and quality) and top-down forces (regulation by predation) in the soil food web [56]. In the present study, microbial biomass increased while the abundances of fungivores and omnivore-predators decreased with N addition. One possibility was that microbial biomass in the temperate steppe was controlled by bottom-up forces, such as the quality of organic material and plant exudates [57], which increased under N additions. Whereas the fungivores and bacterivores were not controlled by bottom-up forces, because fungivores declined even though their resources (total PLFA) increased. Interactions between soil organisms are inherently complex and these bottom-up and/or top-down regulations in the soil food web may also depend on climate and physical conditions.

On the other hand, Bai et al. (2009) found that there is a threshold in the effects of nitrogen addition on aboveground biomass and plant functional composition in the Inner Mongolia grasslands [37]. Based on their findings, we inferred that there may be also a threshold for the N addition effects on the soil micro-food webs. Since high N additions might have a direct toxic effect on some saprophytic fungi by inhibiting their enzymes [58], which might also affect soil nematodes indirectly. SEM analysis also revealed that N addition could affect nematode community through changes in soil pH. Previous reports have suggested that higher N levels are detrimental to omnivores–predators [59], thus precluding any effects of temperature change. Soil acidification or ammonium suppression following N addition has been reported as important factors inhibiting soil nematodes [60].

Corresponding to the decrease in the abundances of fungivores and omnivore-predators, nematode ecological indices such as trophic diversity and generic richness were decreased by N additions, indicating a soil food web with lower diversity in both generic level and trophic community composition. The relatively lower structural and maturity indices under N additions also revealed a degraded food web relative to the treatments without N additions. Due to the trophic complexity associated with soil food webs, our knowledge is still limited about the processes controlling resource acquisition and use in belowground subsystems.

SEM results in this study revealed that the direct effects of N addition on soil food web components were more important than the indirect effects through alterations in soil abiotic characteristics or plant growth. Our observations were not in line with previous findings that changes in nematode community composition operated largely through effects on aboveground vegetation [16], [61]. Since we only tested above- and belowground biomass in the present study, and no significant treatment effects were found on plant biomass, the difference between our study and results by Kardol et al. (2010) and Veen et al. (2010) may be due to the species-specific relationships [16], [61]. De Deyn et al. (2007) found that nematode community composition responded differently to specific plants [62], and previous studies also suggest the important effects of plant species identity on multiple trophic levels in the soil food web [63]. In addition, it is also possible that soil biota may have been affected directly by living plant roots or indirectly through root exudates [64], which were not reflected in the plant biomass during the experimental period (3–4 years). In a long-term biodiversity experiment, Scherber et al. (2010) also found that plant species richness effects generally mediated the belowground responses through changes in root production or root exudates, but not through the vegetation biomass or the amount of litter input [65]. Our results underline suggestions made in other studies that responses of soil biological processes to N addition mainly resulted from direct effects other than indirect effects through changes in soil abiotic characters or plant growth [66], [67].

### Warming effects on soil micro-food web

In our experiment, fungal PLFA biomass decreased following warming. In the same experimental field, the increased temperature associated with climate warming was found to stimulate evapotranspiration, leading to decreases in soil water content and supply of carbohydrates to belowground ecosystems [2], [5]. These changes may help to explain the decrease of fungal PLFA biomass. Similarly, in four heathland ecosystems along a climatic gradient in Europe, experimental warming was found to increase soil microbial biomass at the coldest and wettest site, and decrease soil microbial biomass at the warmest and driest site [40]. While the root-associated fungal community at tundra sites were quite resilient to warming effects [68]. The meta-analysis of 75 manipulative experiments on the responses of soil biota abundance to global change also revealed that effects of warming did not depend on taxon or body size of soil biota, and negative effects of warming were more likely to occur at the colder and drier sites [33].

Warming did not influence other micro-food web components tested in the present study, such as bacterial PLFA biomass or nematode trophic groups. This may be because bacteria and organisms feeding on them are less sensitive to climatic change than fungi, since bacteria mainly live inside soil aggregates and experience less extreme fluctuations in microclimate [69], [70]. In addition, other studies also found that soil moisture mediated the warming effects, thus null and negative effects of experimental warming have been attributed to the reductions in soil moisture [71], [72].

### Interactive effects of warming and N addition on soil micro-food web

One of our objectives in this experiment was to investigate potential interactions between N deposition and climate change. In our study, no interactive effects of warming and N addition were observed on the micro-food web components, which was in support to our hypothesis. Only fungal biomass decreased with warming, so that the absence of interactive effects might be due to the minimal effects of warming on the micro-food web components. Similarly, in a low-alpine heathland, Papanikolaou et al. (2010) also found that there was lack of interactions between warming and N addition [24]. They concluded that N addition and warming acted independently on decomposing and the associated microbial community. Based on our observations in soil micro-food web, the effects of N addition did not depend on warming. Therefore, we conclude that in the temperate steppe ecosystem of our study climate warming and N addition influence soil micro-food web independently.

In summary, the results from our experiment indicated that N addition has led to significant changes in the soil micro-food web of temperate steppe, resulting in a lower diversity belowground micro-food web. However, effects of warming were less obvious than N addition and there were no interactive effects of N addition and warming on soil food web components. Direct effects of N addition on soil food web components explained the observed responses more than indirect effects through changes of soil abiotic conditions.
